# A multi-city urban atmospheric greenhouse gas measurement data synthesis

**DOI:** 10.1038/s41597-022-01467-3

**Published:** 2022-06-24

**Authors:** Logan E. Mitchell, John C. Lin, Lucy R. Hutyra, David R. Bowling, Ronald C. Cohen, Kenneth J. Davis, Elizabeth DiGangi, Riley M. Duren, James R. Ehleringer, Clayton Fain, Matthias Falk, Abhinav Guha, Anna Karion, Ralph F. Keeling, Jooil Kim, Natasha L. Miles, Charles E. Miller, Sally Newman, Diane E. Pataki, Steve Prinzivalli, Xinrong Ren, Andrew Rice, Scott J. Richardson, Maryann Sargent, Britton B. Stephens, Jocelyn C. Turnbull, Kristal R. Verhulst, Felix Vogel, Ray F. Weiss, James Whetstone, Steven C. Wofsy

**Affiliations:** 1 University of Utah, Salt Lake City, UT USA; 2 Boston University, Boston, MA USA; 3 University of California Berkeley, Berkeley, CA USA; 4 The Pennsylvania State University, University Park, PA USA; 5 Earth Networks, Inc., Germantown, MD USA; 6 University of Arizona, Tucson, AZ USA; 7 Jet Propulsion Laboratory, California Institute of Technology, Pasadena, CA USA; 8 California Air Resources Board, Sacramento, CA USA; 9 Bay Area Air Quality Management District, San Francisco, CA USA; 10 National Institute of Standards and Technology, Gaithersburg, MD USA; 11 Scripps Institute of Oceanography, University of California San Diego, La Jolla, CA USA; 12 Air Resources Laboratory, National Oceanic and Atmospheric Administration, College Park, MD USA; 13 Portland State University, Portland, OR USA; 14 Harvard University, Cambridge, MA USA; 15 University Corporation for Atmospheric Research, Boulder, CO USA; 16 GNS Science, Lower Hutt, New Zealand; 17 CIRES, University of Colorado at Boulder, Boulder, CO USA; 18 Environment and Climate Change Canada, Toronto, Canada

**Keywords:** Atmospheric chemistry, Environmental monitoring

## Abstract

Urban regions emit a large fraction of anthropogenic emissions of greenhouse gases (GHG) such as carbon dioxide (CO_2_) and methane (CH_4_) that contribute to modern-day climate change. As such, a growing number of urban policymakers and stakeholders are adopting emission reduction targets and implementing policies to reach those targets. Over the past two decades research teams have established urban GHG monitoring networks to determine how much, where, and why a particular city emits GHGs, and to track changes in emissions over time. Coordination among these efforts has been limited, restricting the scope of analyses and insights. Here we present a harmonized data set synthesizing urban GHG observations from cities with monitoring networks across North America that will facilitate cross-city analyses and address scientific questions that are difficult to address in isolation.

## Abstract (summary)

**Table:** 

Measurement(s)	carbon dioxide • methane • carbon monoxide
Technology Type(s)	spectroscopy
Sample Characteristic - Environment	city
Sample Characteristic - Location	North America

## Background & Summary

Historically, fossil fuel emissions from energy consumed in cities accounted for an estimated 70% of all energy-related GHG emissions^1,2^. Projections of future urbanization trends suggest that the percentage of people living in cities will increase along with urban fossil fuel GHG emissions^1,3^. However, since the adoption of the Paris Climate Agreement in 2016, there has been substantial interest in sub-national greenhouse gas (GHG) mitigation actions, including at municipal spatial scales. Several networks of urban leaders have formed to build momentum and develop best practices for reducing emissions (e.g., C40 cities https://www.c40.org/, Climate Mayors http://climatemayors.org/, and the Global Covenant of Mayors for Climate and Energy https://www.globalcovenantofmayors.org/). The United Nations Framework Convention on Climate Change (UNFCC) hosts an online portal for the Non-state Actor Zone for Climate Action (NAZCA) that include urban actions to address climate change (https://climateaction.unfccc.int/). At the same time as urban leaders have been making commitments to reduce GHGs, new economically competitive technologies are being developed and deployed at scale demonstrating that emission reductions are feasible^4,5^.

With the adoption of urban GHG emission reduction targets, there has been growing interest in monitoring progress and assessing the efficacy of mitigation policies. Creating a simple accounting of GHG emissions using energy consumption data is extremely difficult because granular fossil fuel consumption data are private, have considerable temporal latency, and may not have relevant geographic distributions^6–8^. Research teams have created fossil fuel emission inventories using a variety of techniques, however these can be incomplete or have missing source sectors^8–15^. Urban atmospheric measurements of GHGs are an important complement to these emissions inventories because they are sensitive to “Scope 1” emissions (i.e. direct emissions within an urban airshed) and can therefore be used to evaluate emission inventories^16–18^. Recognising this need, research teams in several cities established urban GHG monitoring networks over the past two decades (Table 1). As these were established, the scientific objectives and network designs varied across cities and research products primarily emanated from the research teams from each city.

**Table 1:** CO_2_-USA urban greenhouse gas monitoring networks.

City	# of sites	Species	City GHG emission reduction target	Project website
Boston^30,31^	8	CO_2_, CH_4_, CO	50%_2005_ by 2030 & net zero by 2050^55^	http://atmos.seas.harvard.edu/lab/index.html
Indianapolis^17,18,32–34^	14	CO_2_, CH_4_, CO	Net zero by 2050^56^	http://sites.psu.edu/influx/
Los Angeles^35^	12	CO_2_, CH_4_	50%_1990_ by 2025, 73%_1990_ by 2035, & net zero by 2050^57^	https://megacities.jpl.nasa.gov/
Washington D.C./Baltimore^46^	13	CO_2_, CH_4_	Baltimore: 15%_2010_ by 2020^58^	https://www.nist.gov/topics/northeast-corridor-urban-test-bed
			D.C.: 50%_2006_ by 2032 & 80%_2006_ by 2050^59^	
Portland^36^	3	CO_2_	50%_1990_ by 2030 & net zero by 2050^60^	http://web.pdx.edu/~arice/CO2_PDX.html
Salt Lake City^37–41^	7	CO_2_, CH_4_	50%_2009_ by 2030 & 80%_2009_ by 2040^61^	https://air.utah.edu/
San Francisco (BAAQMD^*^)^42^	4	CO_2_, CH_4_, CO	Net zero by 2050^62^	https://www.baaqmd.gov/about-air-quality/air-quality-measurement/ghg-measurement
San Francisco (BEACO_2_N^†^)^43^	65	CO_2_	Net zero by 2050^62^	http://www.beacon.berkeley.edu/
Toronto^44,45^	4	CO_2_, CH_4_, CO	30%_1990_ by 2020, 65%_1990_ by 2030, & net zero by 2050^63^	https://www.canada.ca/en/environment-climate-change/services/climate-change/greenhouse-gases-aerosols-monitoring.html

The emissions baseline year is listed in subscript for the city GHG emission reduction targets.

*BAAQMD: Bay Area Air Quality Management District. ^†^BEACO_2_N: Berkeley Environmental Air-quality & CO_2_ Network.

**Fig. 1:**
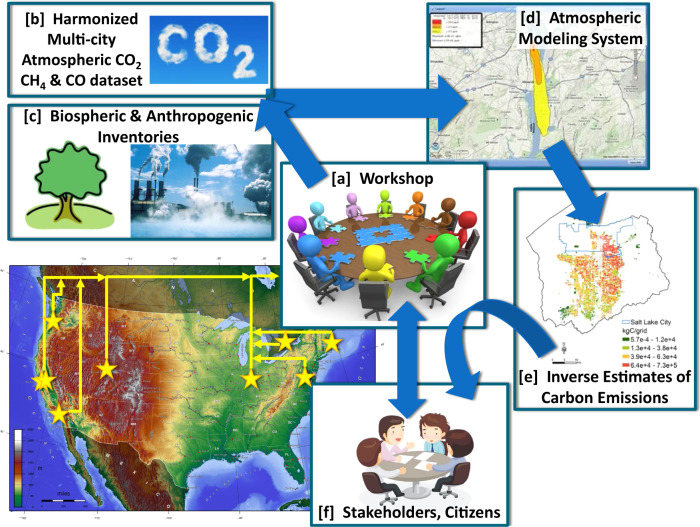
Schematic of the CO_2_-USA project. The map shows the eight cities currently in the CO2-USA network (there are two measurement networks in San Francisco). (**a**) Workshops were held to determine community, stakeholder and policymaker needs. (**b**) This paper describes the harmonized multi-city dataset of atmospheric CO_2_, CH_4_ and CO. (**c**) Inventories of biospheric and anthropogenic emissions were created. (**d**) Updates were made to atmospheric models. (**e**) All of these components can be used together to create estimates of urban carbon emissions using inverse analyses. (**f**) These results can be presented to the community, stakeholders, and policymakers.

The CO_2_ Urban Synthesis and Analysis (CO_2_-USA) network is a synthesis effort primarily supported by the U.S. National Oceanic and Atmospheric Administration, with additional support from National Institute of Standards and Technology. CO_2_-USA was established to build a collaborative network of urban carbon cycle researchers to facilitate data sharing, create analysis frameworks to enable cross-city synthesis analyses^19^, and enable new collaborations tackling objectives that are difficult to address in isolation (Fig. 1). One of the principal objectives of the CO_2_-USA project was to develop a harmonized synthesis data set of atmospheric dry-air mole fraction urban GHG measurements that is readily usable, traceable to international calibration standards, and accessible to a large community: researchers, urban stakeholders, and the public. This synthesis data set could then be compared with remotely sensed or satellite data sets^20–22^ and could also be combined with estimates of biospheric fluxes^7,23^, fossil fuel inventories^14,24–27^, and atmospheric transport models^28^ to evaluate emissions across cities. Building a multi-city analysis framework will enable studies to quantify and understand similarities and differences in how much, where, and why GHG fluxes differ across cities. Applications could include assessing changes in emissions during the COVID-19 pandemic or creating consistent methodologies to assess emissions across different cities that are pursuing emission reduction policies^19^. This information could then be presented to urban stakeholders and policymakers to evaluate progress towards emission reduction goals (Fig. 1). These efforts will also advance similar efforts underway at the international level through the World Meteorological Organization (https://ig3is.wmo.int/).

Here we will present the synthesis GHG mole fraction data product from the CO_2_-USA project incorporating GHG measurements from multiple cities. We will describe the urban monitoring networks, how the data was compiled, how to access it, and how to use it.

## Methods

Construction of the CO_2_-USA synthesis data set follows FAIR data principles^29^, meaning that the data and metadata are Findable, Accessible, Interoperable, and Reusable. The data are findable by having a unique and persistent DOI, and have extensive metadata. They are accessible using standard protocols for retrieval from the web. They are interoperable by using variable naming conventions (Climate and Forecast http://cfconventions.org/ and Attribute Convention for Data Discovery https://wiki.esipfed.org/Attribute_Convention_for_Data_Discovery_1-3) and include citations to the original references documenting the data sets. They are reusable by meeting community standards for carbon cycle measurements with detailed provenance. The data are also distributed with a ‘Fair Use Data Policy’ to guide users on appropriate use and attribution (discussed further in Usage Notes below).

The cities in the dataset include Boston^30,31^, Indianapolis^17,18,32–34^, Los Angeles^35^, Portland^36^, Salt Lake City^37–41^, San Francisco (the Bay Area Air Quality Management District [BAAQMD] network^42^ and the Berkeley Environmental Air-quality & CO_2_ Network [BEACO_2_N]^43^), Toronto^44,45^, and Washington D.C./Baltimore (the Northeast Corridor)^46^ (Table 1). Data providers collected greenhouse gas (GHG) dry-air mole fraction measurements including carbon dioxide (CO_2_), methane (CH_4_), and carbon monoxide (CO) using a variety of instrumentation, experimental setups, and site configurations. All measurements were made using spectroscopy-based instrumentation that includes the Picarro G2301, Picarro G2401, Los Gatos Research Ultraportable Greenhouse Gas Analyzer, Los Gatos Research EP-30, Li-COR 840, LI-COR 6262, and Vaisala CarboCap GMP343. Each of these instruments also measure water vapour to correct for the spectroscopic absorption and dilution of water vapour and reports the mole fraction of the target species on a dry-air mole fraction basis.

Experimental setups varied across cities and sites within cities, but most sites had a gas analyzer, calibration tanks containing reference gas mixtures, a data logger, and tubing to an external inlet^32^. Additional details of the experimental setup and calibrations are described in the Technical Validation section below. The BEACO_2_N network in the San Francisco Bay Area used a modified setup without calibration tanks on site, and instead used a whole network calibration approach to correct for individual instrument drift^43^. Typical site configuration included inlets at each site installed either on towers or on the roofs of buildings. In Los Angeles, Indianapolis, and Washington D.C./Baltimore, several tower sites had up to four inlets at different heights above ground level connected to a single analyzer^34,35,46^. Data providers in each city conducted their own quality assurance and quality control (QA/QC) procedures on the native data set prior to assembling the data in the synthesis data set.

The temporal frequency of the native measurements was another element of experimental design that varied across cities, but each city produced hourly averaged data that is commonly used with atmospheric modelling. In this data product we reported the hourly averages using the “floored hour,” so for example, data from 08:00 to 08:59 were averaged and reported as the hour of 08:00 UTC. In addition, the standard deviation and number of measurements within the hour were reported where possible. Some data providers also calculated a more comprehensive assessment of the analytical measurement uncertainty, incorporating uncertainties from calibration gases, water vapour corrections and other factors^32,35,39,46^. This additional uncertainty was included in the data set where available.

A common approach in the use of urban GHG measurements is to subtract out the mole fraction of the air flowing into the city, usually referred to as the ‘background’ value. The difference between the observed and the background mole fractions results in an important quantity, the enhancement or excess amount that is intended to reflect how fluxes within the city altered the atmospheric composition of the urban air shed. While this is a simple concept, it is complex in practice for several reasons^34,47^, and research groups have used a variety of approaches to construct background mole fractions. The largest difference in approaches is between using a modelled, measured, or hybrid background. Modelled background mole fractions are typically derived by tracking air parcels backward in time and then assigning the air parcel the value from a relatively coarse-scale (~100 km × 100 km) global model such as Carbon Tracker^28,48–50^. Measured background mole fractions can be derived from measurements taken at a measurement site that is upwind of a city^33–35^. A hybrid approach uses measurements and model results to determine a suitable background^31^. An important nuance among these approaches is that the boundary of the city where the background is defined can differ greatly. For example, in a model, the boundary could be 10’s to 100’s of km away from a city depending on the grid spacing within the model. Conversely, an upwind tower could be placed on the edge of a city. For these reasons, the definition of a background is usually dependent on each application.

Despite the diversity in background definitions, it is still useful to report them since they represent an expert assessment of the influence of local processes on the background value. In this data synthesis we have therefore included background values at cities that have presented them in their published literature. For these cases, the city’s data set includes a file listed as ‘background.’ It is important to remember that the background is different from the other measurement sites in the data set, and to refer to the ‘references’ in the file header for additional information about how the background was constructed. For cities without a ‘background’ file, typically one or more of the sites have been used to establish background mole fractions and these details are further described in the city references.

To increase accessibility, the data were archived in both NetCDF (https://www.unidata.ucar.edu/software/netcdf/) and plain text data formats. The text files were created from the NetCDF files to ensure that all of the self-documented header information was identical between the files. In some cities there were multiple inlet heights and multiple species being measured at a single site. This archive used a separate file for each inlet height and species so that the file format was standardized to include a single site location, inlet height, and species in each data file.

## Data Records

**Fig. 2:**
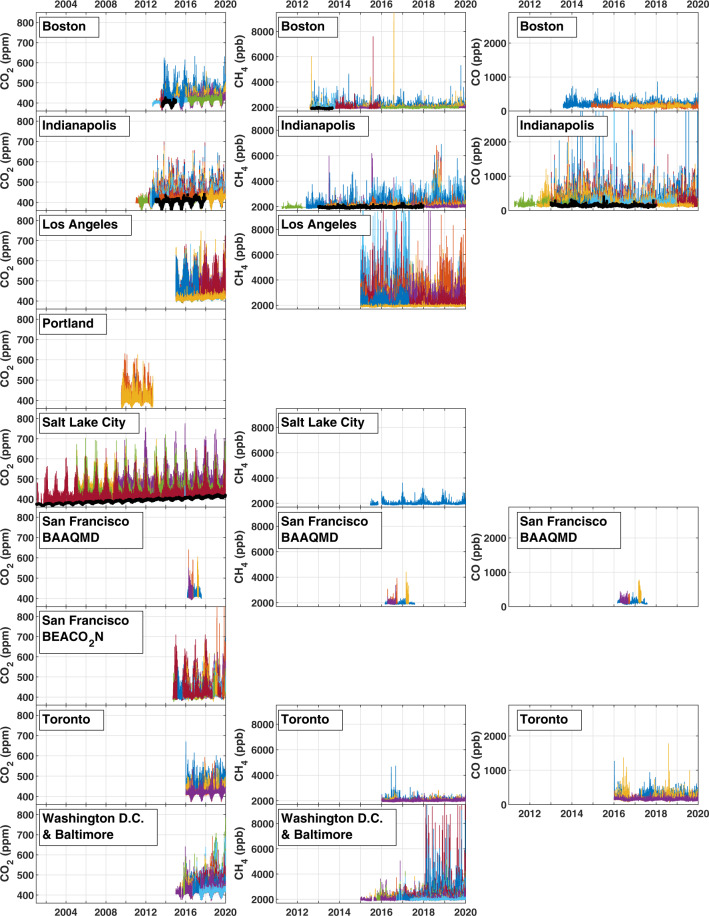
Time series of the CO_2_-USA data synthesis data set. The columns show CO_2_ (left), CH_4_ (middle), and CO (right) while the rows show the CO_2_-USA cities in alphabetical order. Each colored line represents an inlet at a site while the thicker black lines are the background mole fractions (shown for species/cities where a published background is available). BAAQMD: Bay Area Air Quality Management District. BEACO_2_N: Berkeley Environmental Air-quality & CO_2_ Network.

**Fig. 3:**
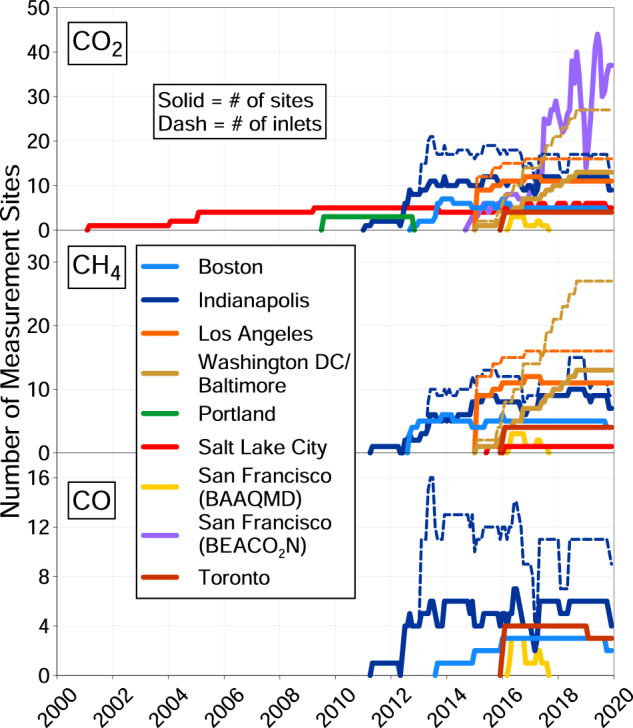
The number of inlets and measurement sites in each city in the CO_2_-USA network over time. BAAQMD: Bay Area Air Quality Management District. BEACO_2_N: Berkeley Environmental Air-quality & CO_2_ Network.

The CO_2_-USA synthesis data set^51^ contains measurements of CO_2_ (671 years of data from 159 inlets at 130 sites), CH_4_ (340 years of data from 78 inlets at 52 sites), and CO (168 years of data from 32 inlets at 22 sites) (Figs. 2 and 3). The code used to build the CO_2_-USA synthesis data files as well as scripts that can extract and plot the data from the data files is maintained on GitHub (https://github.com/uataq/co2usa_data_synthesis). Each data file is generated for a unique site, inlet height, and species, and also contains a comprehensive, self-documented header with the following global attributes that are formatted using the Climate and Forecast version 1.7 (CF-1.7) and Attribute Convention for Data Discovery version 1.3 (ACDD-1.3) conventions:

- Title – A short phrase or sentence describing the dataset.
- Summary – A paragraph describing the dataset, analogous to an abstract for a paper.
- Keywords – A comma-separated list of key words and/or phrases.
- Comment – Miscellaneous information about the data or methods used to produce it.
- References – The original citation where the data, ancillary data, and any uncertainty calculations were presented.
- Source – The method of production of the original data. In this data set all of the data were collected using spectroscopy.
- Date created – The date on which this version of the data was created.
- Date issued – The date on which this data (including all modifications) was formally issued.
- Fair use policy – Fair use policy discussed above.
- Site code – Short code used for the site.
- Site name – Long name description for the site.
- Site latitude – Current latitude of the site.
- Site longitude – Current longitude of the site.
- Site elevation – Current elevation of the site.
- Site inlet height – Current inlet height of the site.
- Site UTC2LST – Number of hours between Coordinated Universal Time (UTC) and Local Standard Time (LST). Note: LST does not include Daylight Savings time offsets.
- Dataset parameter – Species contained in the data file.
- Dataset calibration scale – International calibration scale used for the dataset measurements.
- Dataset start date – Time of the first measurement in the data file.
- Dataset end date – Time of the last measurement in the data file.
- Dataset data frequency and units – 1 hour.
- Provider information – A series of entries about the original data providers including project website, address, and email addresses.
- Compilation information – A series of entries about the data compiler including address and email address.
- ID – Digital Object Identifier (DOI) of the CO2-USA data set.
- cdm_data_type – The data type, as derived from Unidata’s Common Data Model Scientific Data types and understood by THREDDS. (This is a THREDDS “dataType”, and is different from the CF NetCDF attribute ‘featureType’, which indicates a Discrete Sampling Geometry file in CF.) For the CO2-USA data set, this is a “timeSeries.”
- featureType – Description of a single feature with this discrete sampling geometry. For the CO2-USA data set, this is a “timeSeries.”
- Conventions – A comma-separated list of the conventions that are followed by the dataset. For the CO2-USA data set this included: Climate and Forecast version 1.7 (CF-1.7) and Attribute Convention for Data Discovery version 1.3 (ACDD-1.3).

In addition to the global attributes, the following variable attributes were included in each data file:

- UTC time in POSIX format to facilitate a machine-readable time variable.
- UTC time using the ISO 8601 format (e.g., 2016-01-01T00:00:00Z) to provide a human-readable time variable.
- Hourly averaged species mole fractions (e.g., CO_2_, CH_4_, or CO). Hourly averages were reported using the floored hour, so for example, data from 08:00 to 08:59 were averaged and reported under the hour of 08:00 UTC.
- Standard deviation of the native mole fraction measurements within the hour.
- Number of native mole fraction measurements used to calculate the hourly average and standard deviation.
- Uncertainty of the hourly averaged mole fraction measurements determined by the data provider. This variable was included when it was available by the data providers that calculate it. The methods for determining the measurement uncertainty differ between research teams and are not always included.
- Latitude of the site at each hour. This generally stays the same through time, but it can change if a site was moved.
- Longitude of the site at each hour. This generally stays the same through time, but it can change if a site was moved.
- Elevation of the site above sea level at each hour. This generally stays the same through time, but it can change if a site was moved.
- Inlet height above ground level at the site at each hour. This generally stays the same through time, but it can change if a site was moved.

## Technical Validation

Several approaches were used to validate greenhouse gas measurements in each city. At minimum, the research teams in every city calibrated their measurements with working calibration standards in the form of high-pressure gas cylinders that have known mole fractions and are tied to World Meteorological Organization calibration standards, WMO CO_2_ X2007^52^, WMO CH_4_ X2004A^53^, and WMO CO X2014A^54^. Calibration frequency varies across cities and sites from hourly to daily intervals, with the timing being dependent on several factors that could impact instrument drift (e.g., whether or not the instrument is in a temperature-controlled setting). The BEACO_2_N network uses a modified approach with their low-cost sensor network design^43^. That network has a dedicated site with typical calibrations, and then uses a whole-network calibration approach to correct for site-specific instrument drift. In addition to regular calibrations, some cities have conducted ‘round robin’ calibration tests where one or more calibration cylinders were used to test instruments at sites across a city^32^, or between laboratories in different cities^39^.

## Usage Notes

Maintaining greenhouse gas measurement networks requires a substantial amount of expertise and effort, and there are ongoing quality control efforts in each of these GHG measurement programs. While the data set hosted by the ORNL DAAC is openly shared, without restriction, in accordance with NASA’s Earth Science program, we have included a ‘Fair Use Data Policy’ to guide responsible use of the data set:

*These cooperative data products are made freely available to the public and scientific community to advance the study of urban carbon cycling and associated air pollutants. Fair credit should be given to data producers and will depend on the nature of your work. While this data is available under a CC0 license, responsible use includes properly citing the data. When you start data analysis that may result in a publication, we recommend that you contact the data producers directly since they have primary knowledge of their data and any updates and, if it is appropriate, so they have the opportunity to contribute substantively to the analysis and become a co-author. Data producers reserve the right to make corrections to the data based on scientific grounds (e.g. recalibration or operational issues). This dataset is made freely and openly available, with a goal that the results of work using this data also be made freely and openly available to the greatest extent possible*.

To facilitate dissemination and use of the CO_2_-USA synthesis data set, we are maintaining an open access GitHub code repository: https://github.com/uataq/co2usa_data_synthesis. This repository includes the code used to build the CO_2_-USA synthesis data files as well as scripts that can extract and plot the data from the data files. Currently the CO_2_-USA GitHub repository includes scripts in the R (https://cran.r-project.org/), Python (https://www.python.org/), and Matlab (https://www.mathworks.com/) programming languages. Use the following instructions to access the data set:

1. Download the CO_2_-USA synthesis data set^51^ from 10.3334/ORNLDAAC/1916.
2. Download the “co2usa_load_netCDF.r”, “co2usa_load_netCDF.py”, or “co2usa_load_netCDF.m” scripts for use with the R, Python, or Matlab programming language, respectively.
3. Following the instructions in the script, select the cities and species to load, and set the path to the data files on your local computer.
4. Executing the script will load and plot the data.

## Data Availability

All of the code used to create and extract the CO_2_-USA synthesis data set is maintained in an open access GitHub repository: https://github.com/uataq/co2usa_data_synthesis.
